# c-Myc Regulates Self-Renewal in Bronchoalveolar Stem Cells

**DOI:** 10.1371/journal.pone.0023707

**Published:** 2011-08-17

**Authors:** Jie Dong, Shari Sutor, Guoqian Jiang, Yajun Cao, Yan W. Asmann, Dennis A. Wigle

**Affiliations:** 1 Division of General Thoracic Surgery, Department of Surgery, Mayo Clinic, Rochester, Minnesota, United States of America; 2 Division of Biomedical Statistics and Informatics, Department of Health Sciences Research, College of Medicine, Mayo Clinic, Rochester, Minnesota, United States of America; University of Leuven, Belgium

## Abstract

**Background:**

Bronchoalveolar stem cells (BASCs) located in the bronchoalveolar duct junction are thought to regenerate both bronchiolar and alveolar epithelium during homeostatic turnover and in response to injury. The mechanisms directing self-renewal in BASCs are poorly understood.

**Methods:**

BASCs (Sca-1^+^, CD34^+^, CD31^−^ and, CD45^−^) were isolated from adult mouse lung using FACS, and their capacity for self-renewal and differentiation were demonstrated by immunostaining. A transcription factor network of 53 genes required for pluripotency in embryonic stem cells was assessed in BASCs, Kras-initiated lung tumor tissue, and lung organogenesis by real-time PCR. c-Myc was knocked down in BASCs by infection with c-Myc shRNA lentivirus. Comprehensive miRNA and mRNA profiling for BASCs was performed, and significant miRNAs and mRNAs potentially regulated by c-Myc were identified. We explored a c-Myc regulatory network in BASCs using a number of statistical and computational approaches through two different strategies; 1) c-Myc/Max binding sites within individual gene promoters, and 2) miRNA-regulated target genes.

**Results:**

c-Myc expression was upregulated in BASCs and downregulated over the time course of lung organogenesis in vivo. The depletion of c-Myc in BASCs resulted in decreased proliferation and cell death. Multiple mRNAs and miRNAs were dynamically regulated in c-Myc depleted BASCs. Among a total of 250 dynamically regulated genes in c-Myc depleted BASCs, 57 genes were identified as potential targets of miRNAs through miRBase and TargetScan-based computational mapping. A further 88 genes were identified as potential downstream targets through their c-Myc binding motif.

**Conclusion:**

c-Myc plays a critical role in maintaining the self-renewal capacity of lung bronchoalveolar stem cells through a combination of miRNA and transcription factor regulatory networks.

## Introduction

Bronchoalveolar stem cells (BASC) were recently identified in adult mouse lung as a rare cell population located in the bronchoalveolar duct junction [1], [2]. They were identified and purified by FACS based on being positive for Sca-1 and CD34 and negative for CD45 and CD31. This population was also characterized by its double positive staining characteristics for both the alveolar epithelial type II (AT2) cell marker, surfactant protein C (SP-C), and the Clara cell marker, Clara cell secretary protein (CCSP). BASCs demonstrated resistance to naphthalene-induced lung injury and were activated by the oncogenic protein Kras. Viable BASCs were maintained through multiple passages in vitro under distinct culture conditions, and retained the ability to differentiate into either Clara cells or alveolar type II cells AT2 [1], [3], [4]. The observation that almost all cells in the epithelium of the smaller distal airways during early embryonic lung development (E13–E15) express markers of AT2s and Clara cells [5] supports the potential existence of BASCs that are maintained into adulthood once development is complete.

The identification of BASCs has made it possible to begin to map out the pathways that are required for adult progenitor or stem cell function in lung morphogenesis and lung tumorigenesis. Using p38a conditional lung knockout mice, Ventura et al [6] found that inactivation of p38a leads to an immature and hyperproliferative lung epithelium that is highly sensitized to Kras-induced tumorigenesis with coincident expansion of the BASC population. Dovey et al [7] demonstrated that loss of Bmi1 decreases the number and progression of lung tumors with deficient proliferation of BASCs in a Kras-initiated lung cancer model. Yanagi et al [8] generated PTEN knockout mice under control of the SP-C promoter where 90% of mice that received doxycycline in utero died of hypoxia soon after birth. Postnatal deletion of PTEN resulted in spontaneous lung adenocarcinomas with increased BASC numbers. Zhang et al. [9] showed that deletion of Gata6 resulted in the expansion of the BASC population and loss of epithelial differentiation with pronounced activation of the canonical Wnt signaling pathway. These molecular mechanisms regulating the balance between BASC expansion and epithelial differentiation and regeneration suggest that the process of lung tumorigenesis may share critical common pathways with lung organogenesis and response to lung injury.

Stem cells are vital to all stages of life. During early embryogenesis, pluripotent embryonic stem cells (ESCs) differentiate to give rise to the three germ layers that establish the basic vertebrate body plan. As development proceeds, distinct subsets of stem cells emerge to orchestrate the construction of tissues and organs. Once tissues are fully established, stem cells undergo a fundamental change as their role turns from tissue building to tissue maintenance and repair, which persists throughout adult life [10], [11]. The unique ability of ESCs and adult tissue stem cells to self-renew and give rise to multiple cell lineages has formed the basic definitions of “stemness.” We hypothesized that portions of the core transcriptional program of “stemness” present within ESCs may also be shared within putative adult stem cell types such as BASCs. We explored potential underlying mechanisms for maintaining the self-renewal capacity of BASCs by screening stemness transcription factors among 34 genes from a transcription network for pluripotency in ESCs, along with 19 genes from candidates for reprogramming fibroblasts into a pluripotent embryonic stem cell-like state [12], [13]. We found that c-Myc is the most prominent transcription factor upregulated in BASCs, with increased expression in BASCs, Kras-initiated lung tumors, and early lung developmental stages compared with normal adult lung.

Myc is a global transcriptional factor, binding E-box elements (with its partner Max) to regulate gene transcription [14]. Myc can bind to sites covering up to approximately 10–15% of the genome and can regulate thousands of genes. c-Myc is highly expressed in many normal proliferating cells and in cancer. It plays a critical role in regulating cell growth, proliferation, loss of differentiation, and apoptosis [14], [15]. In transgenic mice, targeted overexpression of c-Myc has been shown to be sufficient to induce cancer [16], [17], [18]. In the lung, Myc overexpression in alveolar epithelium results in the development of adenocarcinoma [19], while inhibition of Myc triggers rapid regression of incipient and established Ras-induced lung adenocarcinoma [20]. c-Myc controls two large independent sets of target genes. One set of target genes is transcriptionally regulated by binding of the c-Myc/Max complex to E-Boxes in target gene promoters. Another set of target genes are post-transcriptionally repressed by Myc-induced miRNAs [21].

In this study, we utilized a loss-of-function approach to explore the potential role of c-Myc in the self-renewal of mouse BASCs through a combination of miRNA and transcription factor regulatory networks.

## Results

### BASC isolation and CCA^+^/SP-C^+^ double staining

The synthesis of SP-C is a unique feature of AT2 cells and is commonly used to identify these cells from other lung parenchymal cells. CCA, also known as CC10 or CCSP, has been widely used as a marker for Clara cells. Previous studies have shown that CCA^+^ SP-C^+^ double-positive cells located in the bronchoalveolar duct junction are a putative pulmonary stem cell population in the adult mouse lung. These cells, referred to as BASCs, were originally described as positive for CD34 and Sca1 and negative for CD31 and CD45.

**Figure 1:**
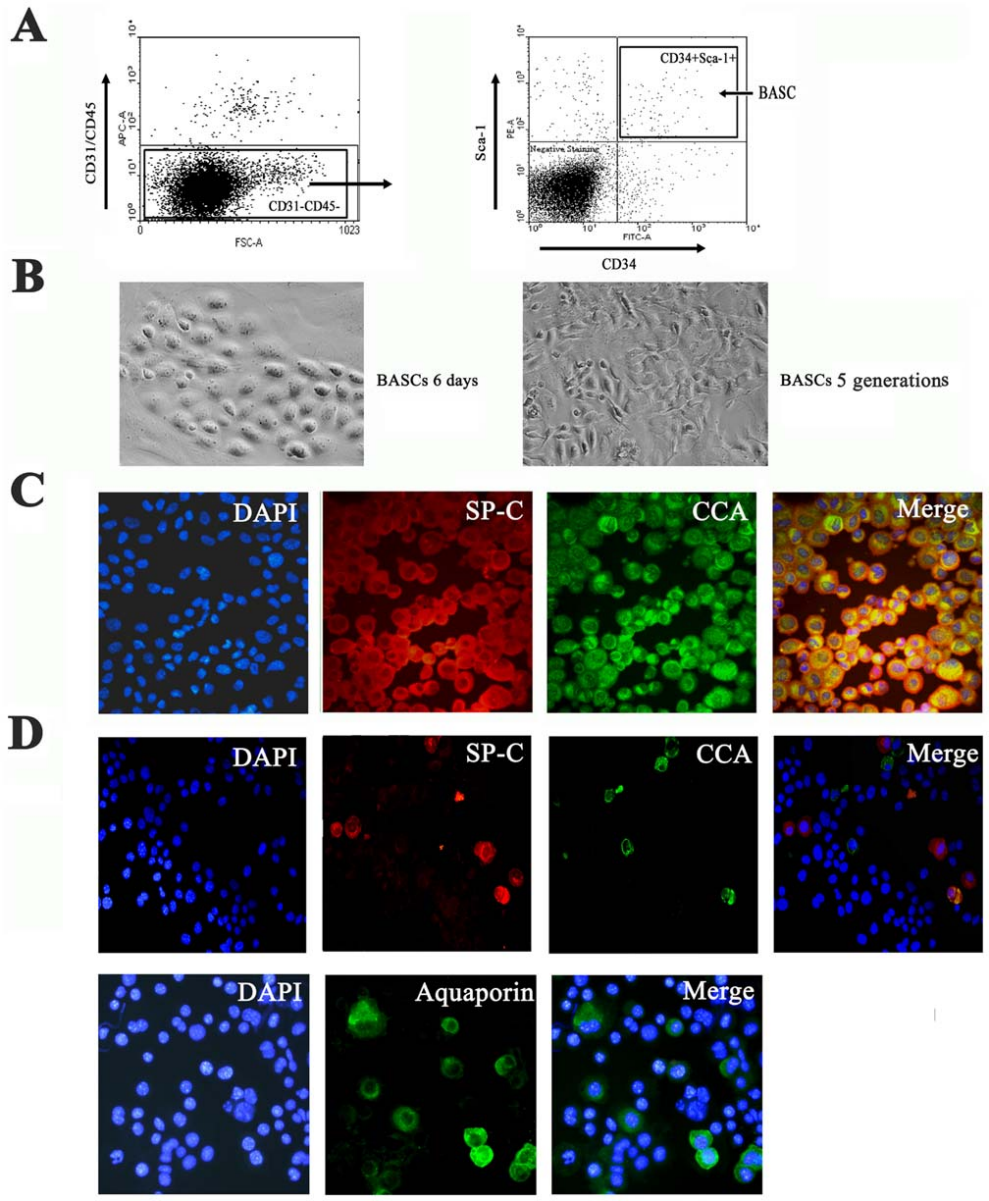
Characterization of BASCs. (A) Methodology of FACS sorting of BASC. First, cells were gated on the CD31^−^ CD45^−^ population, and then subsequently gated on the CD31^−^ CD45^−^ CD34^+^ ScaI^+^ population (BASCs). (B) Phase-contrast images of clonal growth of BASCs on day 6 and BASCs in culture after several generations in serial culture (200×). (C) Identification of BASCs by dual-color immunofluorescent staining for anti-SP-C (red) and anti-CCA (green). (D) Differentiation of BASCs in SAEM media was identified by staining for anti-SP-C (red), anti-CCA (green) and anti-Aquaporin (green).

We used FACS to isolate CD31^neg^CD45^neg^CD34^pos^ScaI^pos^ cells (Figure 1A) and plate onto irradiated MEF cells in BASC media. After the population was established, cells were cultured on collagen-coated dishes and subcultured every 7 days at a density of 1∶20 in BASC media (Figure 1B). To confirm these cells subcultured for several generations still maintain the original marker profile, double immunostaining of CCA and SPC were performed. The expression of both SP-C (red) and CCA (green) were detected in the cytoplasm of the majority of cell colonies, as expected, even after multiple passages in culture (Figure 1C).

In addition to colony formation and proliferation, BASCs retained their capacity for differentiation in serial culture. To obtain differentiation of BASCs, the cells were cultured in SAEM media. Immunophenotyping with CCA, SP-C and Aquaporin-5, a marker of AT1 cells, confirmed the multilineage differentiation capacity of BASCs. Differentiated cells including Clara-like cells (CCA^+^ SP-C^−^), AT2-like cells (SP-C^+^CCA^−^) and AT1-like cells (Aquaporin^+^) were all present with removal of LIF and growing cells in SAEM media (Figure 1D).

### Screening of “stemness” transcription factors in BASCs

Considering the unique ability of embryonic stem cells (ESCs) and adult tissue stem cells to self-renew and give rise to multiple cell lineages, we hypothesized that some or many of the known members of the core transcriptional program for “stemness” would also be present in BASCs. We identified common genes by screening a total 53 transcription factors, including 34 genes from a transcription network for pluripotency of ESCs [12], and 19 genes from candidates for reprogramming fibroblasts into a pluripotent embryonic stem cell-like state including Oct4, Nanog, c-Myc and Sox2 [13].

**Figure 2:**
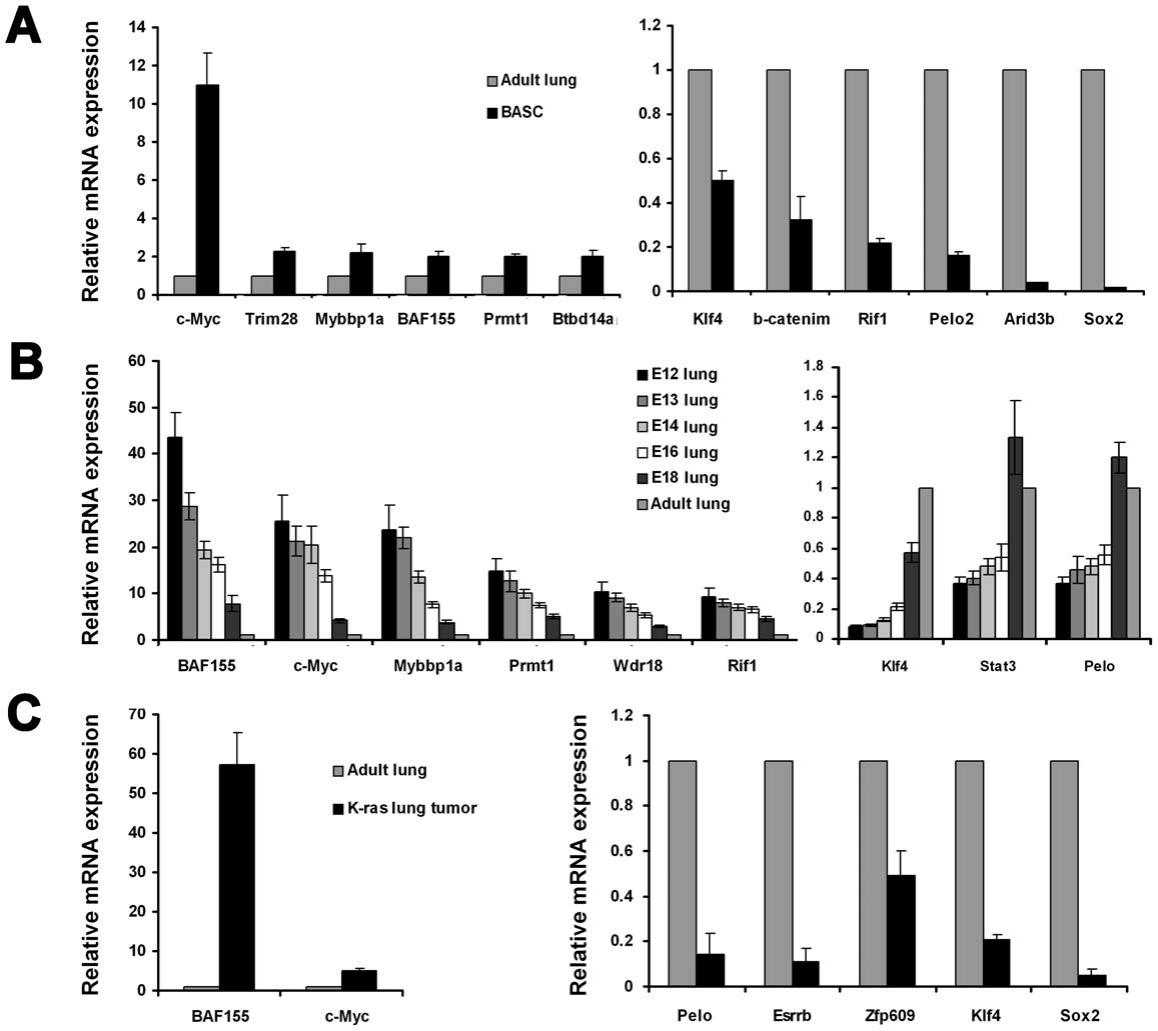
Expression of stem cell transcription factors. (A) In BASCs. (B) In mouse lung in different developmental stages. (C) In K-ras mutant activated lung tumor tissue. In total, expression of 53 stem cell transcription factors were measured including 34 genes from a transcription network for pluripotency of embryonic stem cells [20], and 19 genes from candidates for reprogramming fibroblasts into a pluripotent embryonic stem cell-like state [13] using quantitative real-time PCR. β-actin was used as an internal control. Statistically significant differences are shown. Values are means ± SD of the fold increase compared with adult mouse lung (*n* = 3).

Statistically significant differences in gene expression are shown in figure 2. Compared with normal lung tissue, 6 genes (c-Myc, Trim28, Mybbp1a, BAF155, Prmt1, Btbd14a) were increased and 6 genes (Klf4, β-Catenim, Rif1, Pelo, Arid3b, Sox2) were decreased in BASCs (Figure 2A). BAF155, c-Myc, Mybbp1a, Prmt1, Wdr18, Rif1 were increased, and Klf4, Stat3, and Pelo were decreased during the time course of lung development (Figure 2B). BAF155 and c-Myc were increased and Pelo, Esrrb, Zfp609, Klf4, and Sox2 were decreased in K-ras lung tumors compared with normal lung (Figure 2C). We found that c-Myc was the only transcription factor increased in both BASCs and K-ras-initiated lung tumors, and decreased longitudinally over the time course of lung development.

### Depletion of c-Myc inhibits proliferation of BASCs

**Figure 3:**
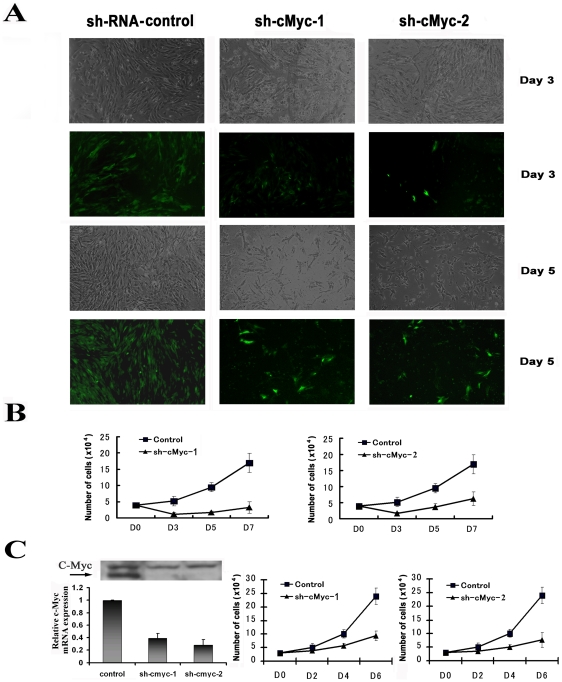
Inhibition of c-Myc expression in BASCs. (A) BASCs were plated in 24-well plates at 40,000 cells per well and incubated in complete medium overnight to achieve 50% confluence. BASCs were infected with viral supernatant including control shRNA or two different c-Myc shRNAs. At day 3, day 5 and day 7 after infection, the cells were washed with PBS and images were taken under both normal light and blue light for GFP expression. (B) BASCs were suspended and stained with trypan blue at the indicated time points. Stained cells were placed in a hemocytometer and viable (unstained) cells were counted. Plotted values are the means ± SD of three replicates. (C) After several generations of puromycin selection, stable c-Myc knock down cell lines were established. c-Myc mRNA and protein expression were measured by real-time PCR and western blotting and cells were counted at the indicated time points.

To determine whether c-Myc is a critical factor for BASC growth and survival, we depleted its endogenous expression by transfecting lentivirus-delivered c-Myc shRNA into BASCs. We observed that many BASCs died within 3 days following transfection (Figure 3A). The number of viable cells was downregulated 58% at day 3, and 62% at day 5 among two different c-Myc shRNA constructs following transfection (Figure 3B). After several generations of puromycin selection, stable c-Myc knock down cell lines were established and the degree of c-Myc reduction in both mRNA and protein level was determined. We found that BASCs infected with control lentivirus continued to proliferate logarithmically; in contrast, cells infected with varying c-Myc shRNAs demonstrated dramatically reduced proliferation rates (Figure 3C).

### Identification of dynamically regulated mRNAs by c-Myc in BASCs

To explore the molecular architecture of the c-Myc pathway in BASCs, gene expression microarray analysis was performed. The log2 expression values of 45,037 probe sets in replicate for two groups (c-Myc depleted BASCs and wild type BASCs) were obtained after normalization. Using a cut-off value of fold change as >|2|, and a *p*-value for ANOVA analysis set as *p*<0.05, 316 dynamically regulated probes representing 250 unique genes were identified. Among these, 131 genes were increased, and 119 genes were decreased in comparing c-Myc knockdown cells with wild type controls (Table S2).

**Figure 4:**
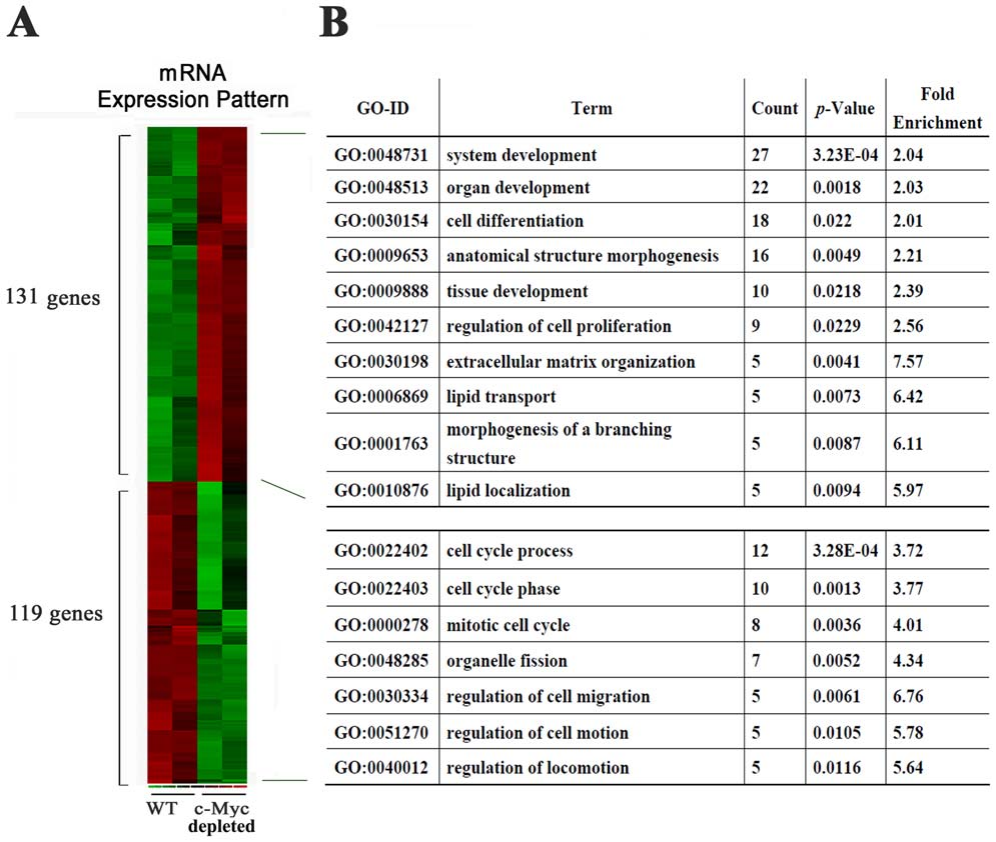
c-Myc-regulated mRNA expression pattern and gene ontology analysis. (A) Heat map of significantly changed genes in BASCs with and without c-Myc. (B). Analysis of enrichment of GO biological process categories for each expression pattern with *p*<0.05 and fold enrichment >2 are listed (ranked by count number).

To focus on the mechanisms that underlie the generation of this c-Myc profile, including the genes induced or repressed by c-Myc, we used the online biological classification tool DAVID to analyze the significant enrichment of these genes for biological processes in GO categories. We listed all GO terms (ranked by count number) in each group with *p*<0.05 and fold enrichment >2. Genes with increased expression in comparing c-Myc knockdown cells with wild type controls were enriched for the terms development and cell differentiation, while genes with decreased expression were enriched for the terms cell cycle and regulation of cell migration and locomotion (Figure 4).

### Identification of miRNAs dynamically regulated by c-Myc depletion in BASCs

**Figure 5:**
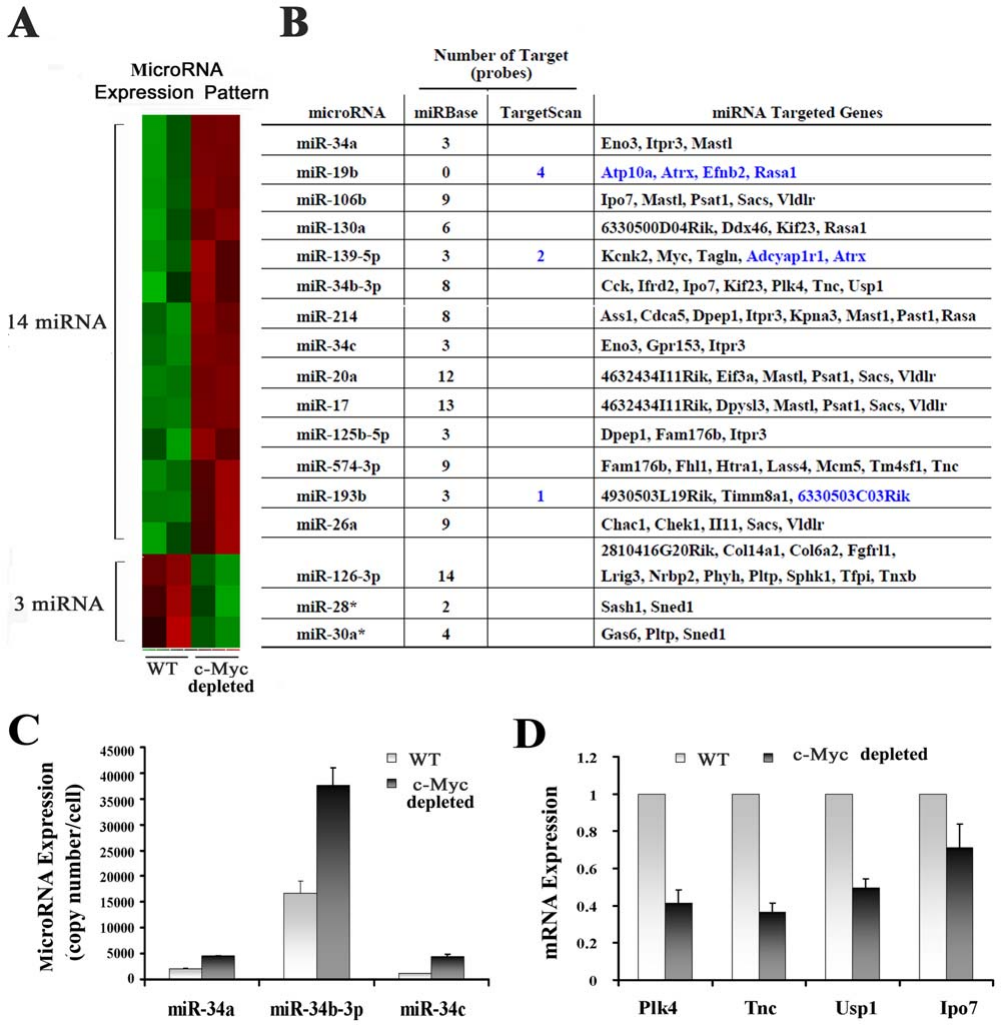
Expression patterns of c-Myc-regulated miRNA and miRNA targets. (A) Heat map of significantly changed miRNAs in BASCs with and without c-Myc. (B) Targeted genes and the genes numbers for each individual miRNA identified through overlapping the potential targets from the miRBase and TargetScan predicted mRNA targets (blue). (C) Effect of c-Myc on the expression of miR-34 family. (D) Relative expression of mRNA targeted by miR-34-3p using real-time PCR (n = 3, mean ± SE).

To address a potential role for miRNAs in a c-Myc regulatory program, miRNA expression profiling was performed using the Taqman real-time PCR based method to screen 521 mouse miRNAs. In total, the expression of 256 miRNAs was detected in control and c-Myc depleted BASCs and their values normalized using an internal control. For the expression data, a cut-off value for Min was set as ΔC_T_<10 and a cut-off value of fold change as >|2|. The *p*-value for ANOVA analysis was set as *p*<0.05. In total, 17 miRNAs were identified through these criteria, the expression of 14 miRNAs were upregulated, while 3 miRNAs were downregulated in comparing the c-Myc knockdown group with wild type (Figure 5A). Several members of the miR-17-92/106b cluster including miR-17, miR-19b, miR-20a and miR106b were increased.

Among these 17 dynamically regulated miRNAs, the top 5 with the greatest fold change were miR-126 (23-fold), miR-34c (17-fold), miR-130a (12-fold), miR-574 (9-fold) and miR-193b (8-fold). The top 5 miRNAs with the highest expression values were miR-19b, miR-125b, miR-17, miR-214 and miR-34b; miR-19b was most substantially expressed with a copy number of 11,333 per cell.

### c-Myc transcriptional regulatory networks in BASCs

#### 1) Identification of target genes regulated by miRNA

Because each miRNA may negatively regulate multiple mRNAs, we investigated whether those significantly changed mRNAs regulated by c-Myc depletion resulted from the regulatory effects of miRNAs. For this analysis, those mRNAs downregulated under c-Myc depletion may be potential targets of an individual miRNA up-regulated and vice versa. We then cross-referenced these potential mRNA targets against the computationally predicted targets of the 17 miRNAs regulated by c-Myc depletion from miRBase and TargetScan. In all, we identified 109 miRNA-mRNA probe pairs, involving 52 unique mRNAs from miRBase, and 7 miRNA-mRNA probe pairs which included 6 unique mRNAs from TargetScan (Figure 5B). Similar to previous results of such mapping for miRNA-mRNAs target pairs in normal lung development [28], this would suggest that the TargetScan database is more stringent with lower sensitivity than miRBase.

In our real-time PCR miRNA array data, miR-34a, miR-34b-3p and miR-34c were increased when c-Myc was depleted in BASCs (Figure 5C). MiR-34b-3p in particular was very highly expressed and dramatically increased after c-Myc depletion. Based on computational predictions from miRBase, there are a total of 11 potential target mRNAs for miR-34 within our data displaying decreased expression. We evaluated a number of these potential targets by real-time PCR. We found Plk4 (Polo-like kinase 4), Tnc (Tenascin C), Usp1 (Ubiquitin specific peptidase 1) and Ipo7 (Importin 7) expression decreased significantly as identified in the array data (Figure 5D). Plk4 is necessary for cellular proliferation, as alteration in Plk4 levels cause significantly adverse mitotic defects including abnormal centrosome duplication and aberrant mitotic spindle formation [29]. In the lung, Tnc appears at the epithelial-mesenchymal interface during branching morphogenesis where it may promote airway branching [30], [31]. Usp1 depletion impacts the stability and phosphorylation of Chk1 which is a cell cycle regulated and DNA damage checkpoint protein [32]. Given their known biological function, the downregulated expression of these genes is consistent with our observation of impaired BASC proliferation induced by c-Myc depletion.

#### 2) Identification of c-Myc/Max binding motifs in c-Myc-regulated mRNAs and miRNAs

A transcription factor protein typically regulates downstream genes through binding via a specific motif on the promoter of its target genes. To ascertain potential direct downstream target genes of c-Myc by identifying its binding sites, a position weight matrix (PWM) based method MATCH (BKL TRANSFAC® Pro) was used to perform sequence similarity searches. We predicted c-Myc binding sites from genomic sequences spanning 2 kb upstream of the transcriptional start site (TSS) through the first exon for all 250 c-Myc-regulated genes we identified in BASCs using the “Best supported” promoter method and p-value<0.01. In total, 208 different c-Myc/Max binding sites were found in 88 gene promoters (Table S2). Although it is possible that c-Myc binding sites may occur outside the regions we studied, the coverage was determined from the knowledge that bona fide c-Myc binding sites appear to cluster within 2 kb of the TSS, and there is a prevalence of c-Myc binding sites in the first intron of many target genes [33], [34].

## Discussion

In this study, we explored the dynamic transcriptional and post-transcriptional regulatory mechanisms responsible for self-renewal in bronchoalveolar stem cells. First, we successfully isolated BASCs by using FACS to sort CD31^neg^CD45^neg^CD34^pos^ScaI^pos^ cells, and demonstrated their ability to maintain double staining of CCA and SP-C with serial passage in culture. Through the hypothesis of a common transcriptional regulatory network in embryonic and other stem cell types, we identified c-Myc as a potential regulator of self-renewal in BASCs. We showed that the depletion of c-Myc in BASCs by infection with Myc shRNA lentivirus resulted in decreased cell proliferation and cell survival rate. Comprehensive miRNA and mRNA profiling was performed, and identified 17 miRNAs and 250 mRNAs significantly changed in expression level with c-Myc depletion. Lastly, we explored a c-Myc transcriptional and post-transcriptional regulatory network by examining a combination of c-Myc direct binding with 5′-promoter sequences, and the role of miRNAs induced by Myc depletion subsequently binding with the 3′-untraslated region (UTR) of target genes.

Myc is necessary and sufficient to maintain self-renewal in mouse embryonic stem cells [35], and together with Sox2, Nanog, Klf4, form an important group of transcription factors capable of reprogramming mouse and human fibroblasts to a iPS (induced pluripotent stem cell) state indistinguishable from known characteristics of embryonic stem cells [13]. Ectopic expression of Myc inhibits the differentiation of ESCs, and inhibition of Myc induces differentiation of ESCs [36]. Myc function in the inhibition of differentiation and promotion of proliferation are present not only in ESCs, but also in adult stem cells. It is hypothesized that the activity of adult stem cells is essential to replenish mature cells constantly lost due to normal tissue turnover. Myc overexpression upsets this balance between self-renewal and differentiation, leading to an expansion of stem cell pools and a concomitant loss of all differentiated cell lineages in hematopoietic stem cells, renal stem/progenitor cells and skin stem cells [36], [37]. In our study, Myc was highly expressed in BASCs, the developing lung, and Ras-induced lung adenocarcinoma. Depletion of c-Myc in BASCs led to decreased cell survival and a reduction in cell proliferation. It remains to be demonstrated if depletion of c-Myc in BASCs will promote differentiation to AT1, AT2 and Clara cells similar to the promotion of differentiation with c-Myc depletion in ES cells. We did observe however that the mRNA expression of differentiated lung epithelial markers including SP-C, CCA and Aquaporin were unchanged in our array data, suggesting that the incomplete depletion of c-Myc alone has no effect on the differentiation of BASCs (data not shown).

**Figure 6:**
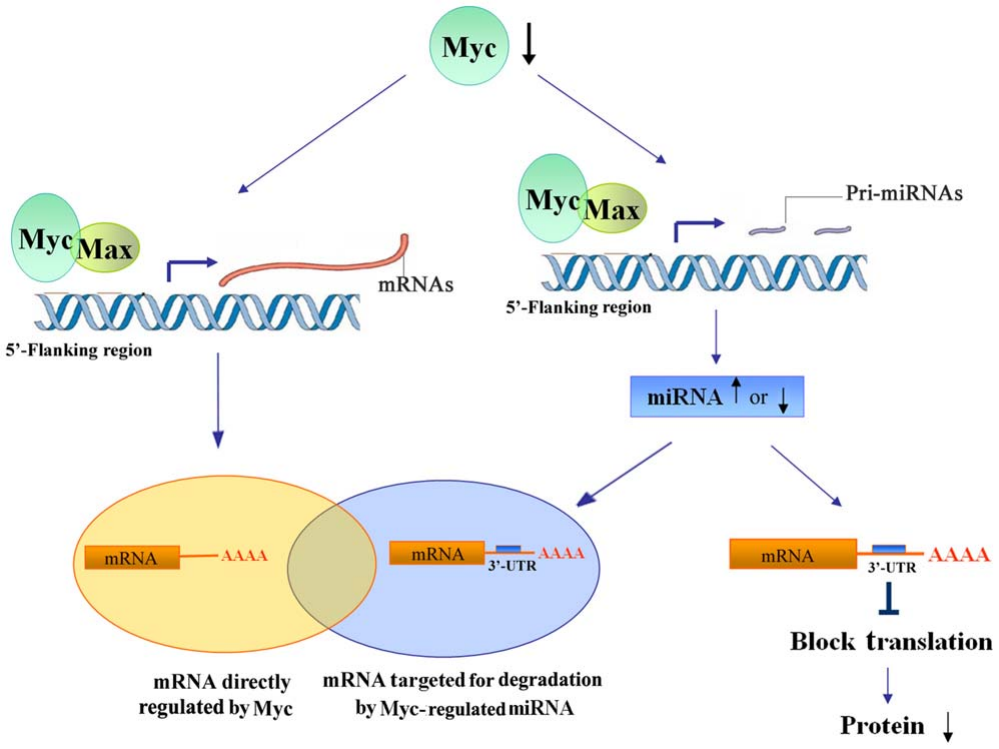
Schematic representation of c-Myc regulating cell proliferation and controlling cell fate in BASCs through both transcriptional and post-transcriptional pathways. The depletion of c-Myc in BASCs resulted in decreased proliferation and cell death. 250 mRNAs and 17 miRNAs were dynamically regulated in c-Myc depleted BASCs. The model (from our work and others) depicts c-Myc regulating downstream gene expression through two distinct mechanisms. 1) Transcriptional level: c-Myc directly regulates mRNA transcription with its partner Max through c-Myc/Max binding sites in the 5′-flanking promoter region of target genes. 88 genes were identified as potential downstream genes through a position weight matrix based method in conjunction with changes in mRNA level. 2) Post-transcriptional level: c-Myc indirectly regulates mRNA and protein expression through miRNAs. Myc can regulate miRNA expression through c-Myc/Max binding sites [41], and we identified Myc-regulated miRNAs by comparing the c-Myc depleted group with controls. The mature miRNA can then negatively regulate gene expression through one of 2 mechanisms; a) by miRNA/mRNA binding and degradation, which is dependent on sequence complementarity between the miRNA and the target mRNA (57 genes were identified as targets of miRNAs through miRBase and TargetScan-based computational mapping); or b) miRNAs suppressing gene expression by blocking protein translation independent of changes in mRNA level [28], [40].

Myc is a transcriptional factor, directly binding E-box elements (with its partner Max) to regulate gene transcription [14]. In our study, we identified 250 Myc-regulated genes in BASC cells. Enrichment analysis of gene ontology terms indicated that downregulated genes in comparing c-Myc knockdown cells with wild type controls were associated with cell cycle and cell migration functions, while upregulated genes were associated with development and cell differentiation. Among these dynamically expressed genes, 88 genes were identified that had at least one binding site for Myc/Max complexes on their respective promoters (Figure 6).

In addition to regulating protein coding mRNAs, accumulating evidence demonstrates that Myc transcriptionally regulates miRNAs [38]. Recent work suggests that Myc regulates a much broader set of miRNAs than previously anticipated [39]. MiRNAs are a class of small RNAs that regulate the expression of target genes at the post-transcriptional level. They are first transcribed from miRNA genes in the nucleus as pri-miRNAs, then transported into the cytoplasm as hairpin structures where they are cleaved to form mature single strand miRNAs. Mature miRNAs are then recruited into nucleoprotein complexes called RNA-induced silencing complexes (RISC). Based on the pairing of miRNAs and their 3′-UTR target sites, the complexes can inhibit translation by either degradation of the mRNAs or by blocking translation without degrading the targets [28], [40].

In our study, through miRNA profiling, we identified 17 Myc-regulated miRNAs in BASC cells in culture. We noticed that several members of the miR-17-92/106b cluster, such as miR-17, miR-19b, miR-20a and miR106b, were increased in expression with depletion of Myc in BASCs. A number of studies have demonstrated that the miRNAs in this cluster play roles in lung development and lung cancer [41], [42]. The potential mechanisms involved may include activation of targets such as RbI2 E2F1-3, and PTEN, all known cell cycle regulators [43], [44]. However, Carraro et al. suggests that the function of the miR-17-92 cluster may be to maintain the structural homeostasis of developing lung epithelium through the targets Mapk14 and stat3, as systematic inhibition of miR-17 did not produce an arrest of proliferation [45]. Through comparison of miRNA and mRNA expression profiles in lung development, previous work by our group indicated that the cluster most likely plays a role in the later stages of lung development after lung branching morphogenesis is complete [28]. Our data from this study support the notion of the miR-17-92/106b cluster having a role on inhibition of proliferation and promotion of differentiation.

The miR-34 family was first identified as transcriptional targets of p53 [46], [47]. MiR-34a is the most well characterized family member and is transcribed from a locus separate from miR-34b/c which is produced as a single primary transcript [48]. In our study, three miR-34 members including miR-34a, miR-34b-3p and miR-34c were increased with Myc depletion in BASCs, supporting the concept that all three members have potent anti-proliferative affects with miR-34a additionally promoting apoptosis [48], [49]. In our study, miR-26a was increased with depletion of Myc in BASCs. It has been reported that miR-26 is transcriptionally regulated by Myc and contributes to tumorigenesis [39].

Computational miRNA target prediction algorithms have been developed based on common features of known miRNAs and their mRNA target interactions. These have greatly facilitated the search for miRNA targets, but computational miRNA target prediction does not account for true cellular context and physiologic factors [50] . For the current study, both miRBase and TargetScan were used to predict direct miRNA targets by comparing negatively correlated miRNA expression with computational predictions. Comparisons with TargetScan require stringent seed pairing, and miRBase requires moderately stringent seed pairing, but even perfect base pair matching does not necessarily guarantee interaction between specific miRNAs and target genes [51]. Wobble base pairs are often tolerated in target sites [52]. Given these differences, we utilized results from both the miRBase and TargetScan databases. In total, we identified 57 mRNA targets for 17 miRNAs through such an approach. Interestedly, we found that 25 genes were overlapping between 57 miRNA-regulated genes and 88 genes which had Myc/Max binding sites on their respective promoter. Of Myc-induced genes, 28% appeared to be regulated by Myc-induced miRNA, suggesting these miRNAs may partially exert their function by acting as rheostats to fine-tune gene expression (Table S2, Figure 6).

In this study, we provide insight to how c-Myc might promote cell proliferation and control cell fate in BASCs through both transcriptional and post-transcriptional means. As a transcription factor, Myc binds to E-box elements with its partner Max and regulates the transcription of genes whose products function to drive cell cycle progression and lung development. In addition to regulating protein coding mRNAs, Myc also transcriptionally regulated non-coding miRNAs with negatively correlated mRNA expression and predicted target biding sites. This finding suggests a potential mechanism where some of this expression is a direct result of Myc binding to miRNA promoters [39], with these miRNAs subsequently modifying mRNA expression on a post-transcription level.

## Methods

### Mice and tissue preparation

Approval of the study protocol was obtained from the Mayo Clinic Institutional Animal Care and Use Committee with protocol number A9807. All mice were maintained in a specific-pathogen-free animal facility at Mayo Clinic and all animal experiments were carried out according to the provisions of the Animal Welfare Act, PHS Animal Welfare Policy, and the principles of the NIH Guide for the Care and Use of Laboratory Animals. Lungs of different development stages were isolated by manual dissection with the aid of a dissecting microscope from timed-pregnant ICR mice. Lung tumor tissue was derived from somatic activation of mutant K-ras in a well-described line of transgenic mice [22].

### BASC isolation and culture

BASCs were isolated from the lungs of 4–6 week old ICR mice according to established protocols [1]. Mice were sacrificed, and their lungs were removed and cut into small pieces. Tissue dissociation was performed by treating with dispase (Invitrogen) at 37°C for 20 minutes, then passing the lungs through a 70 µM filter to obtain a single cell suspension. After washing with PBS (phosphate buffered saline), cells were stained with Biotin-conjugated anti-CD31 and anti-CD45, APC-conjugated streptavidin, PE-conjugated anti-Sca1 (BD Pharmingen), and FITC-conjugated anti-CD34 (eBioscience). Cells were washed and sorted for CD31^neg^, CD45^neg^, CD34^pos^ and ScaI^pos^ cells on an Aria Flow Cytometry machine. Sorted BASCs were plated onto irradiated MEF (mouse embryonic fibroblast) cells with BASC media (DMEM, L-glutamine, MEM Non-essential amino acids, Penicillin/Streptomycin, b-mercaptoethanol, HEPES, fetal bovine serum, and LIF). After the population was been established, cells were cultured in BASC media and passaged every 7 days onto MEFs.

### BASC immunostaining

BASCs were stained with anti-SP-C and anti-CCA (1∶500, Santa Cruz) in PBS with 0.2% triton X-100 overnight at 4°C after cells were fixed and blocked. After washing, BASCs were incubated for 30 minutes with rhodamine conjugated donkey anti-goat (Jackson Laboratories), followed by FITC donkey anti-rabbit antibody for 30 minutes at room temperature. After washing, DAPI nuclear staining was performed. The immunostaining of Aquaporin-5 (Calbiochem), a marker of AT1 cells, was performed after BASCs were cultured in SAEM (small airway epithelial media, CnT-34, CellNted) to allow differentiation for 28 days.

### RNA isolation and assessment of stem cell transcription factors

Total RNA was extracted from mouse embryonic stem cells (R1, generous gift of Dr. Janet Rossant) by using Trizol Reagent (Invitrogen). RNA was reverse-transcribed and standard RT-PCR for the 53 genes described was performed (Invitrogen). PCR products were run on agarose gels and the DNA band was cut and purified using the QIAEX II Gel Extraction Kit (Qiagen). DNA amount was subsequently measured using a DNA concentration standard. Developing lung, lung tumor tissue, and cultured BASCs were washed by PBS and total RNA extracted. After reverse-transcribing, absolute quantitative real-time was performed using Brilliant SYBR Green QPCR Master Mix (Stratagene) and the PRISM 7900 system (Applied Biosystems) according to the manufacturer's instructions. After PCR, melting curves was constructed to ensure elimination of nonspecific products. The amount of mRNA was determined by comparing with the standard curves and normalization by β-Actin. The primer sequences utilized are listed in Table S1.

### Lentiviral constructs and transfection

Mouse c-Myc shRNA lentiviral expression vectors (4 in total) and a negative control vector were purchased from GeneCopoeia. All vectors contained the stable cell line selection marker puromycin and EGFP in the vector backbones. Among the 4 shRNA vectors, 2 vectors which targeted regions beginning at 1705 (ACGTCTTGGAACGTCAGAG) and 1923 (CAGCTTCGAAACTCTGGTG) nucleotides for the c-Myc mRNA sequence (NM_010849.4) demonstrated the strongest inhibition of c-Myc. 293 FT cells were transfected in the presence of 10 ug packaging plasmids (GeneCopoeia) and 2 ug of the Lentivirus expression vectors using FuGENE 6 (Roche). Viral supernatants were collected 48 hr after transfection, filtered through disposable 0.45 um cellulose acetate filters (Millipore) and frozen in individual aliquots at −80°C.

BASCs were plated in 24-well plates at a density of 40,000 cells per well and incubated in complete medium overnight to achieve 50% confluence. BASCs were then infected by removing the culture medium and replacing it with 0.25 ml viral supernatant and 0.25 ml complete medium in the presence of 8 ug/ml polybrene (Sigma) for 24 hours. c-Myc knockdown stable BASC cell lines were generated through puromycin (Sigma) selection.

### Western blot Analysis

Immunologic detection of c-Myc with rabbit polyclonal c-Myc antibody (Cell Signaling, 1∶1000 dilutions) was performed. In brief, 20 µg total protein was loaded on an 8% polyacrylamide gel, electrophoresed, and transferred to polyvinylidene diflouride membranes, which were blocked with Tween-Tris–buffered salt solution (TTBS) containing 5% skim milk. Membranes were incubated overnight at 4°C with c-Myc antibody. After washing 3 times with TTBS, membranes were incubated with anti-rabbit IgG-HRP for 1 hour at room temperature and images recorded.

### mRNA and microRNA expression arrays

Messenger RNA expression profiling was performed using the Affymetrix GeneChip Mouse Genome 430 2.0 Array containing probes to query more than 39,000 transcripts. The reverse transcription, labeling and hybridization of mRNA were performed in the Mayo Microarray Shared Resource.

MiRNA expression profiling was performed using the Taqman Rodent MicroRNA Array Card A and Card B (Applied Biosystems) containing all 521 mature mouse miRNAs in miRBase 10.1 http://microrna.sanger.ac.uk. In brief, miRNA was reverse transcribed to cDNA using the Megaplex TM RT Rodent Primers Pool and the TaqMan MicroRNA Reverse Transcription Kit. Quantitative 384 well TaqMan Low Density Array real-time PCR was run on the ABI PRISM 7900 System using the TaqMan Universal PCR Master Mix. Raw miRNA array data was analyzed using RQ manager software on the ABI system. All undetectable data and the data with C_T_ values greater than 35 were treated as 35 [23]. Normalized C_T_ (ΔC_T_) was calculated by comparing each miRNA value to that of small nuclear U6 RNA. The U6 RNA is a common internal control for each microRNA array card. The copy number of miRNAs in each cell (assuming each cell contains 30 pg of total RNA) was calculated from a formula 10^(40-C^_T_^)/3.34^/22 that was estimated using synthetic lin-4 miRNA [24]. Each profiling experiment is the result of 2 replicates. All array data have been submitted to the Gene Expression Omnibus (GEO) database, with accession number GSE30323 for mRNA and GSE30435 for miRNA data.

### Data processing and analysis

Both mRNA and miRNA array data analyses were performed with the Partek Genomics Suite 6.4 software. For mRNA expression data, Affymetrix CEL files were imported. The data were normalized with the Robust Multichip Average Algorithm [25] and converted to log_2_ values. For miRNA expression data, the ΔC_T_ value was directly imported as a log_2_ value. The greater the ΔC_T_ value, the lower the miRNA expression value. Logged data were used for hierarchical clustering and statistical analysis. After eliminating genes with expression levels at or below background, fold change and p-values derived from ANOVA analysis were used to filter out significantly changed miRNA and mRNA probes.

We assumed that an individual upregulated miRNA may have potential mRNA targets with downregulated expression. Overlapping these potential targets with the computational mRNA targets of each miRNA retrieved from the miRBase (version 5) and TargetScanMouse (5.1) databases, we identified a collection of direct mRNA targets for each miRNA. The software package g:Profiler was used to convert Transcript IDs from miRBase into Affymetrix mouse 430 probe set IDs [26].

### Gene ontology and promoter analysis

Using whole probe sets from the Affymetrix mouse 430 2.0 array as a reference gene list, gene ontology (GO) enrichment analysis was performed using the Database for Annotation, Visualization and Integrated Discovery (DAVID, National Institutes of Health) [27]. The most widely used method for recognition of TF binding sites is the application of positional weight matrices (PWM) [19]. MATCH is a PWM based method from BKL TRANSFAC® Pro that was used for c-Myc binding site analysis.

## Supporting Information

Table S1 **Primer sequences for real-time PCR.**
(XLS)

Table S2 **Significant genes altered by c-Myc depletion in BASCs, potential regulation by miRNA, and presence of Myc/Max motifs.**
(XLS)
